# The Case of Medication-Related Osteonecrosis of the Jaw Addressed from a Pathogenic Point of View. Innovative Therapeutic Strategies: Focus on the Most Recent Discoveries on Oral Mesenchymal Stem Cell-Derived Exosomes

**DOI:** 10.3390/ph13120423

**Published:** 2020-11-25

**Authors:** Amerigo Giudice, Alessandro Antonelli, Emanuela Chiarella, Francesco Baudi, Tullio Barni, Anna Di Vito

**Affiliations:** 1 Department of Health Sciences, University Magna Graecia of Catanzaro, 88100 Catanzaro, Italy; a.giudice@unicz.it (A.G.); antonellicz@gmail.com (A.A.); 2 Department of Experimental and Clinical Medicine, University Magna Graecia of Catanzaro, 88100 Catanzaro, Italy; emanuelachiarella@unicz.it (E.C.); baudi@unicz.it (F.B.); barni@unicz.it (T.B.)

**Keywords:** medication-related osteonecrosis of the jaw (MRONJ), oral mesenchymal stem cells, exosomes, immunity, inflammation

## Abstract

Bisphosphonates-related osteonecrosis of the jaw (BRONJ) was firstly reported by Marx in 2003. Since 2014, the term medication-related osteonecrosis of the jaw (MRONJ) is recommended by the American Association of Oral and Maxillofacial Surgeons (AAOMS). Development of MRONJ has been associated to the assumption of bisphosphonates but many MRONJ-promoting factors have been identified. A strong involvement of immunity components has been suggested. Therapeutic intervention includes surgical and non-surgical treatments, as well as regenerative medicine procedures for the replacement of the lost tissues. The literature confirms that the combination of mesenchymal stem cells (MSCs), biomaterials and local biomolecules can support the regeneration/repair of different structures. In this review, we report the major open topics in the pathogenesis of MRONJ. Then, we introduce the oral tissues recognized as sources of MSCs, summing up in functional terms what is known about the exosomes release in physiological and pathological conditions.

## 1. Introduction

It’s widely accepted that cell-to-cell communications take place via both cell contacts and release of paracrine factors. Paracrine factors in particular could be released as soluble factors, protein complex and packaged mediators [1,2]. In the case of mesenchymal stem cells (MSCs), it becomes evident that tissue regeneration capability is mainly dependent on paracrine effects such as the secretion of trophic factors, cytokines and extracellular vesicles (EVs).

EVs are phospholipid bilayer membrane-enclosed structures which can be secreted by the majority of cell types and then released in extracellular space and fluids. EVs are classified on the basis of both vesicle size and biogenesis in four categories: exosomes, released via exocytosis with the size of 30–150 nm; microvesicles, released via budding with the size of 100–1000 nm; retrovirus-like particles, 90–100 nm, which appear similar to retroviral particles and contain a portion of retroviral protein; apoptotic bodies, released during apoptotic events and generally larger than 1000 nm [3,4]. More recently, a different type of vesicles smaller than 50 nm has been characterized and termed exomeres [5,6]. EVs can be generated by a variety of pathways which generally accounted for the release of a heterogeneous populations of EVs, which differ for size as well as for composition and functions.

Aside from these aspects, many attempts have been made in order to characterize the exact composition of EVs, however it’s clear that their composition results extremely variable and is cell- and environment-dependent. Indeed, proteomic and genomic complexities of EVs are still scarcely recognized. MSC-derived EVs composition appears even less characterized in pathological conditions.

Medication-related osteonecrosis of the jaw (MRONJ) has been recognized as one of the most disabling comorbidities associated to the assumption of bisphosphonates (BPs). In the last decades, researchers investigate on the pathogenesis of MRONJ and potential therapeutic approach. The most intriguing and promising tool results to be the application of undifferentiated cells able to regenerate and restore the destroyed tissues. Mesenchymal cells recovered by different sources such as bone marrow, dental pulp, periodontal ligament have been applied in experimental therapeutic approaches both as cell suspension or cell sheet. MSCs have also been employed in combination with biocompatible scaffolds. The evidence that MSCs administration contributes to tissues restoring above all via the release of paracrine factors shift the attention to the exact nature and composition of such soluble factors [7].

In this review, we attempted to summarize the most recent discoveries about the composition of exosomes released by oral MSCs. We reported the major open topics in the pathogenesis of MRONJ, suggesting new experimental approaches in this field. Then, we introduced the different oral tissues recognized as source of MSCs, summing up in functional terms what is known about the exosomes release in physiological and pathological conditions.

## 2. Pathogenesis of Bisphosphonates-Related Osteonecrosis of the Jaw

Osteonecrosis of the jaw is defined by at least three pathognomonic signs: (1) exposed bone in the maxillofacial region that does not heal within 8 weeks after the identification; (2) exposure of the patient to an antiresorptive agent; (3) no history of radiation therapy to the craniofacial region [8]. The first case of MRONJ was reported by Marx in 2003 [9]. Initially, osteonecrosis was reported only after treatment with BPs and then named bisphosphonate-related osteonecrosis of the jaw (BRONJ); since 2014, the term medication-related osteonecrosis of the jaw (MRONJ) was recommended by the American Association of Oral and Maxillofacial Surgeons (AAOMS) [10]. Indeed, accumulating evidence suggested the involvement of other antiresorptive and antiangiogenic treatments among the causes of MRONJ, such as bevacizumab, sunitinib, aflibercept and denosumab [11,12,13,14]. More recently, the administration of other antiangiogenics such as dasatinib, erlotinib, imatinib, axitinib, sorafenib and cabozantinib has been associated with MRONJ [15]. A detailed review of the drugs related to the development of MRONJ has been recently published [15]. Today, the nitrogen-BPs (N-BPs) alendronate and zoledronate, and the human monoclonal antibody targeting the receptor activator of nuclear factor kappa-Β ligand (RANKL), denosumab, have been suggested as drugs most commonly associated to the development of MRONJ.

The antiresorptive effects of N-BPs and denosumab are different. Briefly, N-BPs are analogs of pyrophosphate able to bind to the hydroxyapatite crystals of bone. Upon incorporation within osteoclast, they inhibit the enzyme farnesyl pyrophosphate (FPP) synthase and decrease prenylation of low molecular weight-proteins [16,17]. Moreover, the inhibition of FPP-synthase accounts for the accumulation of isopentenyl diphosphate (IPP), the production of a toxic metabolite (ApppI) and cell apoptosis [18]. Conversely, the monoclonal antibody denosumab, approved by the U.S. Food and Drug Administration (FDA) in June 2010 for the treatment of osteoporosis in postmenopausal (PM) women with a high fracture risk [19,20], displays the capability to bind and inhibit RANKL reducing bone resorption. Importantly, BPs accumulate in the skeleton for a long time, in contrast with denosumab whose half-life is approximately 26 days [21]. The most recent classification or staging system for MRONJ has been proposed by the AAOMS. Briefly, patients were assigned to different stages according to the criteria reviewed by Ruggiero in 2014, and then classified as “at risk”, stage 0, stage 1, stage 2 characterized by the appearance of infection and stage 3.

What is the trigger for the MRONJ? Although the development of MRONJ has been associated to the assumption of N-BPs, many MRONJ-promoting factors have been also identified, such as dental treatments and pre-existing oral infections. In the past, many experiments have been carried out in order to investigate the effects of N-BPs and non-N-BPs on the development of MRONJ. Most of the research focused on their pro- and/or anti-inflammatory action, showing both overlapped and opposed effects. To this purpose, typical experimental model of in vitro inflammation requires the administration of lipopolysaccharide (LPS), recognized as a major anaerobic bacteria component able to trigger inflammatory cascade in oral tissues. Indeed, oral cavity harbors many microenvironments constitutively exposed to microorganisms which are directly or indirectly involved in periodontal diseases such as periodontitis and MRONJ [22]. In particular, a state of deficit healing process and reduced vascular supply of the jaws is a favorable condition to establish an infective disease like osteomyelitis or MRONJ, caused by commensal bacteria of the oral cavity [23]. Moreover, the loss of oral mucosal integrity and submucosal infections increases the infection risk by saprophytic bacteria of the oral cavity which in basal conditions have low virulence [24,25]. Several studies indicated *Actinomyces spp.* as one of the main players in MRONJ onset, however, other bacteria typical of the oral microbiota like *Streptococcus spp.* and aciduric bacteria play an important role in the bone colonization [26,27]. Furthermore, as evidenced in a clinical study by Zirk et al., the majority of the oral bacteria isolated in necrotic bone samples was quite different from bacteria isolated from the submucosal infected samples. A high *Actinomyces spp.* presence was detected in the submucosal infections, instead *Viridansstreptococcae*, *Parvimonasmicra*, *Prevotella*, and *Veillonella* species were identify both in bone and soft tissue samples. Usually an anaerobic bacteria dominance was noticed in the bone and submucosal infections, followed by facultative anaerobic gram-positive bacteria [28].

Regarding the cellular and molecular mechanisms involved in the development of MRONJ, the reduced vascularization observed in BPs-treated patients has been suggested as the key player for the necrosis of the jaw. However, as suggested by Dodson, infections-associated lesions often precede the development of necrosis of the jaw, so we can conclude that events occurring during the establishment of the infection result as the trigger for the exacerbation of MRONJ [29]. To date, the order by which infection, inflammation, and inhibition of angiogenesis appear during the pathogenesis of MRONJ remains to be elucidated.

## 3. The Effects of BPs on Cells Residing in the Bone

BPs exert their anti-resorptive action targeting bone-resorbing osteoclasts. However, in the last two decades, many evidence challenges the long-held dogma that BPs act only in the skeleton, showing direct or indirect effects not only on osteoblast, osteocyte, fibroblast and epithelial cells [30,31,32,33,34,35,36], but also on cancer cells and tumor-associated macrophages (TAMs). The molecular mechanisms involved in N-BPs interaction with cells other than osteoclasts have been investigated recently. As a matter of the fact, there are still no robust evidence suggesting that endothelial cells, osteoblast and immunity cells can internalize N-BPs. During bone remodeling that strongly occurs in alveolar bone, osteoclasts are the only cells that are able to release N-BPs via the production of HCl accounting for bone dissolving. In this acidic environment, N-BPs result lipophilic and then able to go across the cell membrane [37]. Nevertheless, Rogers et al. suggested that free BPs or BPs complexed to bone particles or matrix proteins are internalized by osteoclasts into membrane-bound vesicles by endocytosis [16]. It’s interesting to note that after the release of BPs into osteoclast cytosol, free BPs may accumulate in the cytosol or migrate by transcytosis in the extracellular space for their recycling [38]. In neutral environment, N-BPs must be taken into cells by specific transporters such as phosphate transporters. N-BPs enter in the osteoclasts via transporters belonging to the solute carriers (SLC) 20 and/or SLC34 family; non-N-BPs enter via transporters of SLC17 family [39]. There is evidence suggesting that similar to osteoclasts, also TAMs take in BPs by endocytosis or by phagocytosis of BPs bound to microcalcification [38,40,41].

It’s generally accepted that a major event during the establishment of bacterial infection in MRONJ, is the suppression of the immunological reactions; the immunosuppressive state occurs as a result of BPs action on the different cell populations in the oral microenvironment, such as macrophages, γδ T-cells, endothelial cells [42].

### 3.1. BPs-Macrophages Cross-Talk

Inflammation and necrosis have been identified as two hallmarks of N-BPs action [43,44,45]. Some evidence suggested that such effects depend on the N-BP concentrations: inflammation becomes evident at lower concentration while necrosis appears as a consequence of higher drug concentrations. Among the cell populations residing in alveolar socket, macrophages play a key role in the conservation of tissue homeostasis by regulating both the development and the maintenance of inflammation. Since 1993, it became evident that some BPs are able to interfere with immunity response, via direct effects on macrophages, granulocytes and osteoclasts in vivo [46]. N-BPs inhibited monocytes/macrophages release of cytokines, osteoclast commitment, and dendritic differentiation [47,48,49]. More recently, it was described that N-BPs are able to regulated macrophage polarization. Macrophages have been classified into M1 and M2 subtypes: M1 macrophages are functionally pro-inflammatory, while M2 macrophages are anti-inflammatory. N-BPs are able to inhibit M2-polarization and stimulate the pro-inflammatory M1 phenotype both in vivo and in vitro [50,51]. Molecular mechanisms involved in such phenomena have been partially investigated. Macrophages may be stimulated by both damage-associated molecular pattern (DAMPs) and pathogen-associated molecular patterns (PAMPs) which trigger inflammatory responses through innate immune receptors, such as TLRs, and downstream pathways such as nuclear factor-κB (NF-κB).

**Figure 1:**
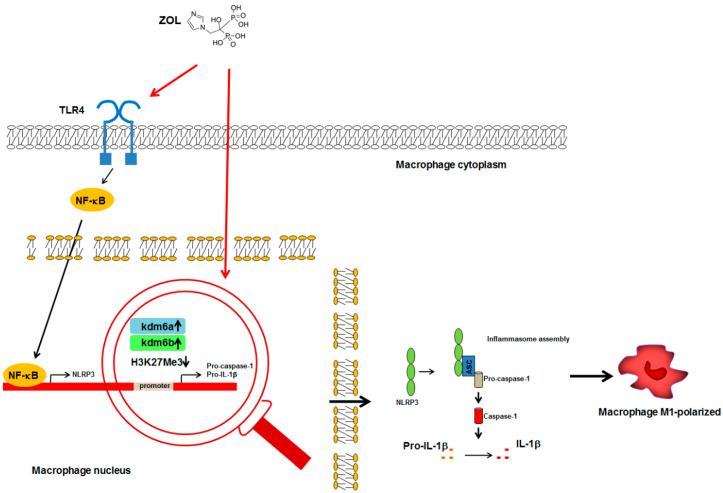
Schematic representation of ZOL-macrophage cross-talk mechanisms. ZOL-mediated activation of TLR4 in macrophages accounted for the overexpression of mature form of IL-1β leading to M1- polarization. Red arrows indicate the action of ZOL on TLR4 activation and Kdm6 proteins overexpression. (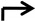): gene transcription; (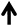): increase; (H3K27Me3 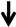): reduced methylation; all the other arrows indicate the direction of the flow; IL-1β, interleukin-1β; Kdm6a, lysine demethylase 6 A; Kdm6b, lysine demethylase 6 B; NF-κB, nuclear factor-κB; NLRP3, NACHT, LRR, and PYD domains-containing protein 3; TLR4, Toll-Like Receptor 4. See text for explanation [51,53,54].

Zhu et al. showed that zoledronate in vivo administration accounted for Toll-Like Receptor 4 (TLR4) activation, NF-κB translocation in the nucleus and M1-polarization of macrophages in the alveolar socket after teeth extraction (Figure 1) [51]. Moreover, it’s known that generally such stimuli promote the assembly of NACHT, LRR, and PYD domains-containing protein 3 (NLRP3) inflammasome complex, the activation of caspase-1 and the production of mature form of interleukin (IL)-1β and IL-18 (Figure 1) [52].

The role of IL-1 signaling in N-BPs-mediated inflammation has been described [53]. Both the two isoforms of IL-1, IL-1α and IL-1β are produced as a 31 kDa precursor which can be converted to proper 17 kDa mature form via enzymatic cleavage. The overexpression of caspase-1 and IL-1β reported in zoledronate treated cells seems to also involve epigenetic mechanisms. Indeed, zoledronate treatment increases the expression of two related JmjC-domain-containing proteins, lysine demethylase 6 A (Kdm6a) and Kdm6b, leading to demethylation of the caspase-1 and IL-1β promoters and their overexpression (Figure 1) [54]. Interestingly, N-BPs-associated IL-1β overexpression is not always accompanied by its overproduction; such effect is due to the fact that caspase-1 expression might not be stimulated by N-BPs [53].

Similar mechanisms have been suggested to be involved in the N-BP-induced fever; indeed, fever occurs among patient receiving the first BP treatment, while does not occur in patients receiving second or repeated intravenous N-BPs. In the latter case, the inhibition of osteoclast, recognized as a major source of IL-1β, accounts for normal or reduced IL-1β level [53,55]. Together, these observations indicate a key role of IL-1β in the N-BPs-induced acute immune response.

### 3.2. BPs-γδ T Cells Cross-Talk

**Figure 2:**
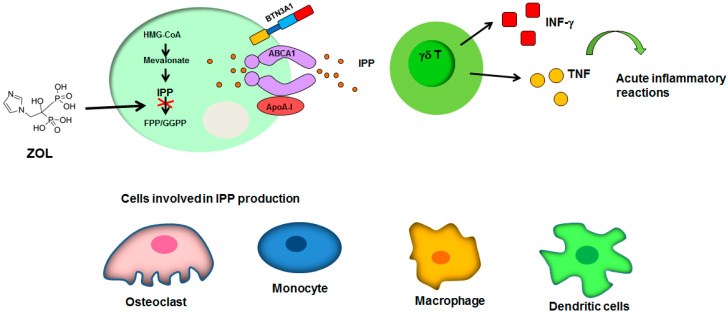
Mechanisms of γδT-cells activation. The ZOL-mediated overproduction of IPP in osteoclasts accounts for γδT-cells activation and triggers acute inflammatory reaction. The cells involved in IPP production also included monocytes, macrophages and dendritic cells. Black arrows indicate the direction of the flow; green arrow indicates the activation of acute inflammatory response. 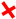: inhibition of FPP synthase. ABCA1: ATP-binding cassette transporter A1; ApoA-I: apolipoprotein A-I; BTN3A1: butyrophilin-3; FPP: farnesyl pyrophosphate; GGPP: geranylgeranyl diphosphate; HMG-CoA: Hydroxymethylglutaryl-CoA Reductase; INF-γ: interferon-γ; IPP: isopentenyl diphosphate; TNF, tumor necrosis factor. See text for explanation [16,56,57,58,59,60,61].

The mechanisms by which N-BPs inhibit osteoclast also account for the establishment of both inflammatory and necrotic events. The most relevant involves the production of IPP during the inhibition of FPP synthase in osteoclasts. Indeed, IPP stimulates VγVδ2 T-cells to produce interferon (INF)-γ and tumor necrosis factor (TNF) triggering acute inflammatory reactions [56,57]. Dendritic cells, monocytes and macrophages-release of IPP seems to also mediate the activation of γδ T-cells [16,58,59]. IPP extrusion is mediated by ATP-binding cassette transporter A1 (ABCA1) in cooperation with butyrophilin-3 (BTN3A1) and apolipoprotein A-I (ApoA-I) (Figure 2) [60,61].

A lot of papers have reported that patients treated with N-BPs showed a significant alteration of γδ T-cells population in peripheral blood despite a higher level of infiltrating γδ T-cells in the lesion area [62,63,64,65]. In particular note, zoledronate was reported to activate T cell surveillance in both bone niche and blood, together with antiangiogenic and immunomodulatory mechanisms [66]. The fact that single or repeated administration of BPs may lead to different results is remarkable [67]. Regarding the molecular mechanisms involved in γδ T-cells-activation, N-BPs seem to induce the release of soluble Sema4D which further stimulates macrophages-release of TNFα. Further studies are required in order to clarify this point.

The mechanisms by which N-BPs account for MRONJ establishment also involve other cell types. Administration of N-BPs and in particular of zoledronate accounts for reduced recruitment of neutrophils and reduced activity of natural killer (NK) cells [68]. During bone remodeling, osteoclasts stimulate NKs cells which, in turn, produce INFγ inhibiting osteoclastogenesis. Osteoclast suppression induced by N-BPs accounts for NK cells deregulation and the maintenance of an immunosuppressive microenvironment, delaying wound healing [69].

### 3.3. BPs-Vascular Endothelium Cross-Talk

N-BPs have been shown to inhibit angiogenesis both in vivo and in vitro, hindering the mobility of cells involved in the immune response [70]. It becomes evident that the inhibitory action towards osteoclast interferes with normal angiogenesis, due to the role of osteoclasts in the correct patterning of the vessels in the bone [71]. Moreover, N-BPs exert their role in inhibiting angiogenesis through both direct and indirect effects. First of all, N-BPs such as zoledronate inhibit endothelial cells and endothelial progenitor cells proliferation, migration and differentiation, accounting for cells apoptosis [68,72]. On the other hand, Ohlrich et al. showed overexpression of vascular-endothelial growth factor (VEGF) in gingival fibroblast after exposure to zoledronate, suggesting the induction of a proangiogenic environment [73]. Similar contrasting effects may be understood in light of the fact that N-BPs alter VEGF receptors maturation in endothelial cells, so that the increased expression of VEGF does not exert any effect [74]. In addition, zoledronate treatment may inhibit angiogenesis by reducing the expression of osteopontin (an angiogenesis inducer) in both maxilla and mandible [75]. Finally, N-BPs may also interfere with endothelial differentiation of mesenchymal cells [76].

## 4. Current and Emerging Treatment Options

During the years several authors have discussed MRONJ treatment. However, while the objective seems to be to minimize the risk of MRONJ onset, unfortunately this is not always possible. In 2014, the American Association of Oral and Maxillofacial Surgeons provided some guidelines in the treatment of MRONJ. They argued that each MRONJ stage should be treated with a specific therapy; stage 0 (patients without any clinical evidence of necrotic bone, but with non-specific symptoms or clinical and radiographic findings) should be strictly monitored and eventually treated with medication for chronic pain and infections; stage 1 (patients with exposed necrotic bone or fistulae without any evident infection) should benefit from medical management with topic antimicrobial and chlorhexidine 0.12% rinses, however immediate surgical treatment is not suggested; stage 2 (patients with exposed necrotic bone or fistulae and clear signs of infection) should be treated with a combination of oral antibiotics, antibacterial mouth rinses and debridement of necrotic areas to relieve soft tissues irritation and to control infection spread; stage 3 (patients with exposed necrotic bone or fistulae, clear signs of infection and huge necrotic bone areas, pathological fractures, extra-oral fistula, oral antral/oral nasal communication and osteolysis process extended to the inferior mandibulae border or sinus floor) should be managed with antibiotics and pain control, antibacterial mouth rinses and surgical debridement/resection of all necrotic zones. However, there is no clarity about the best treatment to prevent or to manage MRONJ. Several authors affirmed as medical therapy with a wide spectrum antibiotic prophylaxis is a vital part to reduce and to manage the symptomatology of MRONJ [77]. However, for the advanced stage of MRONJ is mandatory a combined protocol of antibiotics administration and surgical approach [78]. Although in the past it has been highlighted how conservative surgical treatment can be useful to control bone necrosis progression in the early stages of MRONJ [79], recently it has been observed that radical surgical treatment of these stage is more effective in reducing the recurrence of MRONJ pathology [80]. Furthermore, many innovative tools have been developed to perform a better management of surgical phases: the use of piezoelectric surgery, low-laser therapy and VELscope^®^ device to distinguish the limits of necrotic bone [81,82,83]. Several authors assessed the efficacy of the application of platelet-rich fibrin, and transplantation of autologous bone mixed to MSCs to facilitate a faster tissue healing after surgery [84,85,86,87].

In the last decade, attention was focused on the possible effect of MSCs in the healing of wound caused by BPs [88]. In particular, the first case of MRONJ successfully treated with autologous stem cells transplantation has been reported by Cella et al. [89]. Classically, tissue engineering approach involves scaffolds, growth factors and MSCs, used either separately or in combination in order to restore functional tissues. In this context, it’s important to take in considerations the fact that oral MSCs are influenced by BPs administration, which accounts for the impairment of their capability in tissue repair and osteogenic differentiation [90,91].

## 5. An Overview of the Oral MSCs Ontogenesis

The site specificity of MRONJ has been associated to intramembranous formation of maxilla and mandible, which was responsible for the different histology of jaw respect to long bones. Moreover, jaw displays highest bone turnover according to the majority of researches, and surgical interventions account for increase bone remodeling [92]. Jaw is particularly susceptible to bacterial infections compared to other bones, due to the anatomy of mucosal barrier which appears very thin and vulnerable. Finally, given the fact that jaw derived from neural crest cells (NCCs), it appeared that specific molecular pathways might drive both the differentiation of such structures and the pathogenesis of the jaw [93]. A rapid overview of jaw and teeth development may contribute to the understanding of the pathogenesis of the MRONJ. Briefly, during embryonic development, bone formation occurs by two distinct mechanisms: endochondral ossification and intramembranous ossification. Both mechanisms begin with the formation of mesenchymal condensation. During endochondral ossification, mesenchymal nodule forms a cartilage matrix and mesenchymal cells differentiate in chondroblasts; conversely, during intramembranous ossification mesenchymal nodule differentiates directly in osteoblasts, without chondrogenic phase. Craniofacial skeleton predominantly develops through intramembranous ossification involving NCCs-derived progenitor cells [94]. NCCs give rise not only to bone but also to cartilage and periodontium, via reciprocal interactions between the epithelium and the mesenchyme [95].

After their formation, at the interface between the surface ectoderm and the roof plate of the neural tube, NCCs undergo to epithelial-mesenchymal transition. Thereafter, they migrate to different sites. In particular, NCC responsible for maxilla and mandible formation migrate from midbrain and hindbrain (rhombomeres 1 and 2) through the first pharyngeal arch; teeth morphogenesis arises from the sixth week of development and go through four phases: thickening stage, bud stage, cap stage and bell stage. Thereafter, the development of cementum, alveolar bone and periodontal ligament (periodontium) completes tooth morphogenesis before the eruption. Moreover, the Hertwig epithelial root sheath (HERS) deteriorates and the residual cells form clusters termed as the epithelial rests of Malassez. In the erupted teeth, neural crest cells give rise to different cell types: dental pulp stem cells (DPSCs), dental follicle stem cells (DFSCs), stem cells from apical papilla (SCAPs), stem cells from exfoliated deciduous teeth (SHEDs) and periodontal ligament stem cells (PDLSCs). These cells maintain both self-renewal and differentiation properties [96]. Furthermore, several studies proved that many factors can influence oral MSCs behavior, such as the source, donor age and cell culture conditions [97]. Accordingly, we have previously described the influence of particular cell culture conditions and differentiation factors on both proliferative and osteogenic potential of PDLSCs [98].

## 6. Exosomes

Although the endo-exocytosis intracellular pathways represent a bulky component of classic cellular biology, in the last decades the attention has been directed to intracellular vesicles and particularly to exosomes. The rapid evolution of knowledge in this field is proven by the challenging in the nomenclature of the different types of cellular vesicles [4]. Great interest derived by the discovery of this cellular activity that must not be considered part of a simple waste disposal, but somewhat represents a specific cellular function whose comprehension is still underway. So, the different functions of EVs could open to an evolving number of diagnostic and therapeutic applications. Initially, the different EVs was classified on the basis of biogenesis, charge or size into exosomes, microvesicles and apoptotic bodies. The exosomes, the more relevant EVs with regard to this review, so defined by Johnstone in 1987, appear in cryo-electron microscopy as round structures made up by a double layer membrane. Exosomes originate as intraluminal vesicles (ILVs) via inward budding of the limiting membrane of maturing endosomes, which are usually referred to as multivesicular endosomes (MVEs) or multivesicular bodies (MVBs) [99,100,101].

Depending on the cell of origin and specific physiological or pathological conditions, the exosomes can contain nucleic acids (DNA, RNA, most frequently miRNAs but also other species long noncoding RNA, circular RNA), lipids, proteins, and metabolites. Cells release exosomes in extracellular medium, where they exert several biological effects on neighboring cells or cells that are significantly distant from the place of production. Quantification and characterization of EVs and exosomes appear not easy, and the results obtained in the different experimental settings may vary, depending on the variety of separation and concentration methods, impacting also the functional studies. For these reasons, the International Society for Extracellular Vesicles (ISEV) provides some guidelines for the analyses of these vesicles useful to adopting the best practice [3]. Some databases are also available to the researchers, such as VESICLEPEDIA (http://www.microvesicles.org) or the ExoCarta [102,103].

For many years, EVs release has been considered the mechanism by which cells maintain cellular homeostasis, by the clearance of cellular undesirable and toxic material. Currently, a growing number of evidence of their involvement in many physiological and pathological situations pointed to EVs as mediators of intercellular communication. These pleiotropic functions involve all the principal biological pathways: gene expression regulation, proliferation, apoptosis, differentiation, development, immune response, signal transduction, migration and metabolism regulation. As a consequence, EVs deregulation has been implicated in several disorders from cancer to neurodegenerative diseases. This multifunctional property depends on the highly heterogeneity of the sources and the cargo content [104]. Moreover, it was suggested that cells secrete distinct populations of exosomes with unique size, protein and RNA composition, which display differential effects on the gene expression programs in recipient cells [105].

Although the endosomal theory resulted to be the most prevalent hypothesis on exosomes biogenesis, some evidence suggested a shared pathway from plasma membrane and endosomes [106,107]. The endocytic pathway is organized in key sorting stations in the endosomal system, which are represented by the early endosomes (EEs) and late endosomes (LEs). Transport from EEs to LEs is also accompanied by major proteins and lipids remodeling [108]. During their path, EEs can fuse each other or with vesicles containing acid hydrolases, thus forming intermediate structure, called MVB and containing ILVs which eventually fuse with the LEs. The MVBs could be view as a crossroad containing one population of ILVs with different fates because the cargo can be packaged into lysosomes for degradation, but can also be routed toward other destinations. In particular, ILVs can be released extracellularly as exosomes. The cargo molecules could also be alternately recycled back to the plasma membrane or to the trans Golgi network to retrograde transport. Moreover, the vesicles trafficking inside these dynamic compartments is accompanied by membrane fusion in selected group of heterotypic and homotypic organelles by fusion and fission, so that the exchange and redistribution of the cargo are also possible [109]. In this context, it has been suggested that the specific targeting of the organelles could be useful in the characterization of EVs [108].

The molecular mechanisms regulating the complex EVs trafficking are still incompletely understood [108]. The biogenesis of MVBs is regulated from several processes, likely coexisting and redundant, otherwise interconnected leading to the production of a different subpopulation of ILVs. This has been comprehensively reviewed by [101] and [110]. As pointed out by Palmulli, four major checkpoints can be identified in the process leading to EVs production [101]. The first checkpoint in MVBs production regards the expression of the specific protein cargo; so, the availability of specific proteins in the cell of origin appears crucial for the production of EEs. Of course, the expression of proteins cargo is both cells- and environment-dependent. The internalization of the protein cargo take place via either clathrin-dependent or clathrin-independent endocytosis. The second checkpoint regards the fate of EEs, which can be recycled to the plasma membrane or directed to MVBs. This process is regulated by the postsynaptic density protein, disc-large, zonulin-1(PDZ) protein syntenin, which together with the endosomal sorting complex required for transport (ESCRT) accessory protein ALIX and thesyndecans account for exosomes release. At this point, MVBs also include proteins derived from the Golgi apparatus. In order to reach the plasma membrane for exosomes release, MVBs need to avoid the alternative fusion with lysosomes or autophagosomes. In particular, the ubiquitination status of the proteins plays a key role in the determination of proteins fate [110].

Most of these processes require the participation of ESCRT machinery. In particular, four biochemically distinct protein complexes (ESCRT-0, -I, -II, and -III) have been characterized to play a key role in the maturation of EEs in LEs together with accessory proteins [19]. In addition to ESCRT machinery-dependent pathway, other mechanisms seem to be involved in exosomal release. In particular, a syndecan/sintenin- and a lipid rafts-dependent processes could act in parallel or separately to recruit exosomal cargoes and generate ILVs [111]. The transport of ILVs on the cytoskeleton to the plasma membrane can be considered as the last major checkpoint in the release of exosomes. This process is mediated by molecular motors, such as dynein and kinesin, small GTPases and specific SNARE complexes [110].

### 6.1. Exosomes in Oral Mesenchymal Cells

MSCs can be readily isolated from human oral tissues like dental pulp, apical papilla, periodontal ligament, gingiva, dental follicle, and others [96]. Among them, neural crest-derived MSCs, such as periodontal ligament and dental pulp, achieved greater attention due to specific and important properties associated to their easily harvesting and propagation, multipotential capabilities, and the absence of ethical concerns. Therefore, MSCs have been recognized as a therapeutic tool in the regenerative medicine field, according to their high ability to regenerate tissues from different origin [7]. In addition, MSCs transplantation results in low engraftment rate, so their therapeutic effects might be related to the secretion of soluble mediators acting in a paracrine fashion. In this scenario, the application of oral MSCs-derived exosomes might assume a crucial role as therapeutic approach in the future also for patients with MRONJ. The major literature available regarding the isolation and characterization of exosomes from different sources of oral MSCs is reported in Table 1. In the following sections, authors describe the most recent discoveries on oral MSCs-derived exosomes.

### 6.2. Exosomes Derived from Human Dental Pulp Stem Cells (DPSCs)

**Figure 3:**
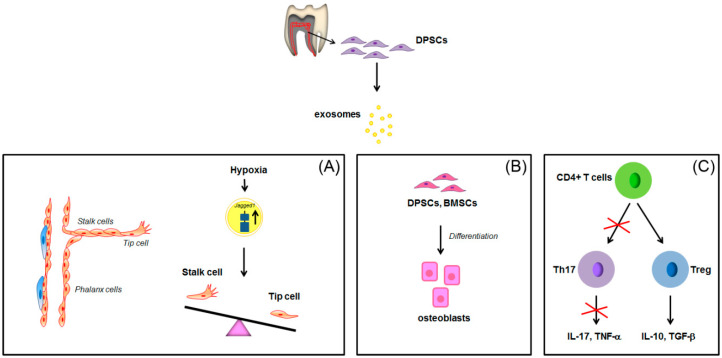
Schematic representation of the paracrine effects of exosomes derived from DPSCs in angiogenesis, osteogenic differentiation and CD4+ cells alternative differentiation. Jagged1 overexpression in hypoxic DPSCs-derived exosomes antagonizes Dll4 in Notch binding, promoting angiogenesis and increasing the number of tip cells (**A**). Exosomes derived from DPSCs induce osteogenic differentiation of DPSCs and BMSCs (**B**) and induce Treg differentiation of CD4+ cells (**C**). 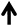: overexpression; 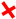: inhibition of Th17 differentiation; all the other arrows indicate the direction of the flow. BMSCs: bone marrow stem cells; CD4+: positive for CD4 expression; DPSCs: dental pulp stem cells; IL-17: interleukin-17; IL-10: interleukin-10; TGF-β: transforming growth factor β; Th17: T helper 17 cells; TNF-α, tumor necrosis factor α; Treg: regulatory T cells. See text for explanation [113,114].

To date, a limited number of papers have described the isolation, composition and function of DPSCs exosomes [112,113,114,115,116,117,118,119,120]. Recently, a proangiogenic action for DPSCs-derived exosomes has been demonstrated. DPSCs-derived exosomes are efficiently taken up by HUVECs and are able to induce cell proliferation and tubule formation via the up-regulation of angiogenesis-related molecules and the involvement of p38 MAPK pathway [116]. Regarding the mechanism by which pro-angiogenic action occurs, Gonzalez-King et al., in 2017 showed the recruitment of Jagged-1, the only Notch ligand present in the exosomes derived from human dental pulp MSCs [114]. It’s known that Notch signaling plays a key role in angiogenesis through its regulation of the balance between tip cells and stalk cell formation during sprouting process, in which Dll4 and Jagged1 ligands display opposing roles. They showed that HIF-1α-overexpressing MSCs secrete higher level of exosomes than MSCs, with an higher concentration of Jagged-1 able to induce angiogenesis both in vivo and in vitro (Figure 3A) [114].

DPSCs-derived exosomes were tested for their capability to induce lineage specific differentiation of naïve MSCs [113]. It appeared that DPSCs-derived exosomes triggered odontogenic differentiation of both DPSCs and BMSCs. Moreover, the exosomes derived from cells cultured in the presence of odontogenic differentiation media were more potent in inducing odontogenic differentiation, most probably as consequence of altered genetic and protein exosomal cargo (Figure 3B) [113]. DPSCs-derived exosomes also induced BMSCs migration and proliferation [120]. Similar results were confirmed by Hu et al., 2019. They characterized the microRNA expression profiles of exosomes derived from DPSCs under odontogenic conditions and observed a set of differentially expressed miRNA involved in the regulation of Transforming Growth Factor β1 (TGFβ1)/SMADS signaling pathway [119]. It’s noteworthy the fact that dental tissue-derived exosomes can potentially direct their action to all cell populations present in the microenvironment. As a matter of the fact, immunosuppressive properties of MSCs are also mediated by exosomes [118]. Ji et al. showed that DPSC-derived exosomes displayed stronger immunomodulatory capability than bone marrow MSCs (BMMSC)-derived exosomes when co-cultured with peripheral blood mononuclear cells [117]. Indeed, DPSCs-derived exosomes inhibited the differentiation of naïve CD4+ T cells into T helper 17 cells (Th17), with the consequent reduction of IL-17 and TNF-α production. At the same time, Treg cell differentiation appeared to be promoted, followed by an increased release of the anti-inflammatory factors IL-10 and TGF-β (Figure 3C). However, also in this case, the specific proteins, miRNAs, mRNAs or lipids involved in this process remain to be elucidated, by the application of both standards methodologies as well as new proteomic methods or bioinformatic platforms [121].

Recently, DPSC-derived exosomes have also been characterized for their capability to regulate migration and differentiation of Schwann cell, recognized as neural-crest-derived stem cells able to regenerate odontoblasts [122]. Exosomes derived from LPS-treated DPSCs accelerated Schwann cell proliferation and migration, via the strengthening of directional cell movement, as well as odontogenic differentiation, suggesting a role for Schwann cells in the regenerative processes [117,122]. DPSC-derived exosomes also displayed neuroprotective efficacy in an in vitro excitotoxicity model of neurodegeneration. In their comprehensive study, Venugopal et al. compared the neuroprotective effects using three strategies: hippocampal neuron-MSC co-culture, neuron-MSC condition medium treatment and neuron-MSC exosomes treatment in an in vitro model of kainic acid-induced excitotoxicity. They showed that neuroprotective action of DPSCs-derived exosomes was similar to that of co-culture strategy and conditioned medium treatment strategy [115].

### 6.3. Exosomes Derived from Human Periodontal Ligament Stem Cells (PDLSCs)

**Figure 4:**
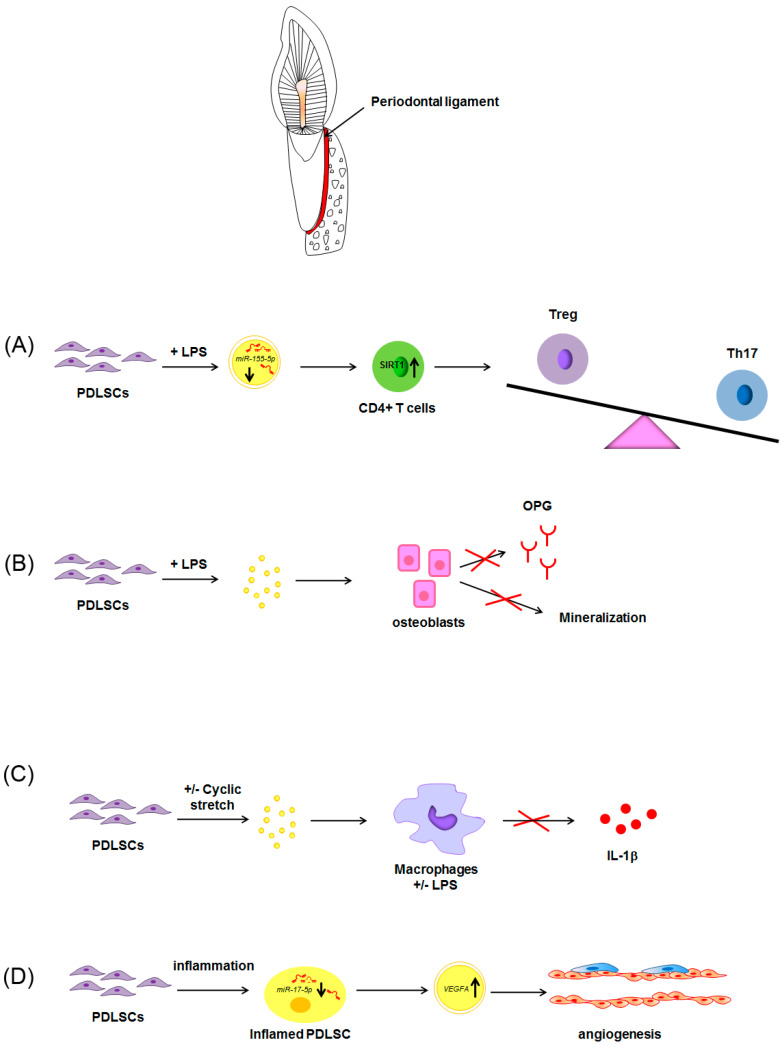
Schematic representation of the paracrine effects of exosomes derived from PDLSCs in CD4+ cells alternative differentiation, osteogenic differentiation, IL-1β production and angiogenesis. Exosomes derived from LPS-treated PDLSCs account for the upregulation of Th17 and the downregulation of Treg phenotype when transferred in CD4+ T cells culture (**A**). Exosomes derived from LPS-treated PDLSCs also accounted for the inhibition of osteogenic activity and osteoprotegerin expression in osteoblasts (**B**). PDLSCs under cyclic stretch release exosomes able to inhibit macrophage release of IL-1β (**C**). Inflamed PDLSCs release exosomes enriched in VEGFA, leading to increased angiogenesis (**D**). 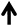: increase; 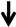 decrease; 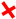: inhibition of the process; all the other arrows indicate the direction of the flow; +/−: plus or minus; CD4+: positive for CD4 expression; IL-1β: interleukin-1β; LPS: lipopolysaccharide; OPG: osteoprotegerin; PDLSCs: periodontal ligament stem cells; SIRT-1: NAD-dependent deacetylase sirtuin-1; Th17: T helper 17 cells; Treg: regulatory T cells; VEGFA: Vascular endothelial growth factor A. See text for explanation [113,114]. See text for explanation [124,126,127,128].

An even lower number of papers have investigated the cargo of exosomes of PDLSCs. Rajan et al. first isolated exosomes from PDLSCs. Subsequent papers showed that PDLSCs-derived exosomes played a key role in events occurring during inflammation in periodontitis [123]. Exosomes derived from PDLSCs are involved in the altered balance of Th17/Treg reported in periodontal tissues of patients with periodontitis. Indeed, although PDLSCs in normal environment accounted for the maintenance of Th17/Treg balance, LPS-treatment of PDLSCs in vitro was responsible for a decreased expression of miR-155-5p in exosomes and a consequent up-regulation of SIRT-1 when transferred in CD4+ T cells culture. The increased expression of SIRT-1 accounted for the increase of Th17 differentiation and the downregulation of Treg phenotype (Figure 4A) [124]. Moreover, exosomes derived from PDL fibroblasts exposed to LPS accounted for the inhibition of osteogenic activity and osteoprotegerin expression in osteoblasts (Figure 4B). From these observations appear clear that PDLSCs-derived exosomes play a key role in bone remodeling that occurred during periodontal inflammation [125].

Recent evidence characterized the anti-inflammatory role of PDLSCs-derived exosomes in PDLSCs-macrophages cross-talk during both physiological and pathological conditions [127]. Exosomes released from PDLSCs under cyclic stretch, that resembles occlusal stimuli, were responsible for the suppression of IL-1β production via the inhibition of the NF-κB signaling pathway. Moreover, PDLSCs-derived exosomes were also able to inhibit NLRP3 inflammasome signaling in human macrophages primed with LPS by inhibiting the NF-κB signaling pathway (Figure 4C) [127]. Also in this case, PDLSCs-derived exosomes play a key role in the maintenance of periodontal immune/inflammatory homeostasis.

As pointed out by Wang et al., the fact that PDLSCs are the only ones among oral mesenchymal cells showing cyclic stretch-induced exosome secretion is noteworthy [127].

Aberrant angiogenesis resulted as pathognomonic sign of periodontitis. Interestingly, PDLSCs played a crucial role in similar event, according to the up-regulation of VEGFA, recognized as the most potent agent participating in modulation of vascular endothelium. According to Zhang et al., inflammation was responsible for miR-17-5p down-regulation in PDLSCs which, in turn, accounted for increased expression of VEGFA in exosomes derived from inflamed PDLSCs. The exosomes-mediated transfer of VEGFA to endothelial cells finally contributed to the increased vascularization of periodontal ligaments in periodontitis (Figure 4D) [128].

Immunomodulatory action of PDLSCs-derived exosomes was also supported by the in vivo research of [129]. They isolated exosomes/microvesicles from PDLSCs of patients showing relapsing-remitting multiple sclerosis, the most common form of multiple sclerosis with 85% of occurrence [130], and showed the capability of exosomes/microvesicles to inhibit NLRP3 inflammasome activation, exerting a protective role in experimental autoimmune encephalomyelitis mice model of multiple sclerosis. Similar effects were reported for conditioned media of PDLSCs of patients showing relapsing-remitting multiple sclerosis.

### 6.4. Exosomes Derived from Human Exfoliated Deciduous Teeth Stem Cells (SHEDs)

Stem cells from exfoliated deciduous teeth are considered as advantageous stem cells source due to the fact that they represent a more immature populations of postnatal stem cells with high proliferation rate and osteoinductive capacity [131]. Exosomes have been successfully isolated by SHED and characterized for their capability of enhancing PDLSCs osteogenic differentiation via the Wnt/β-catenin and BMP/Smad signaling pathways [132].

**Figure 5:**
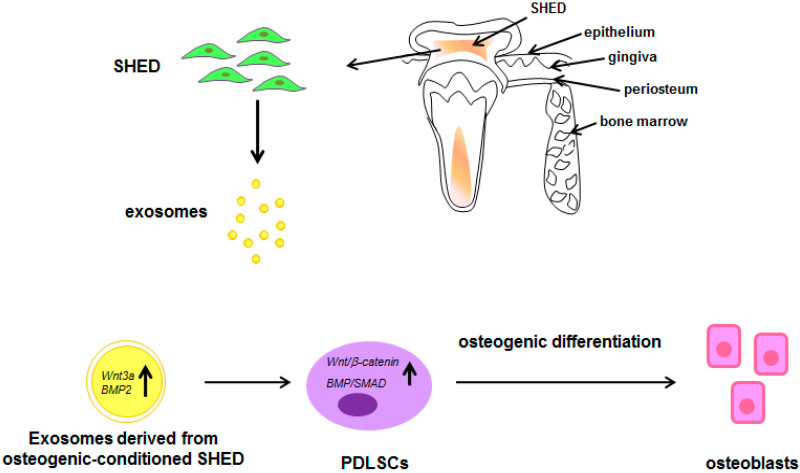
Schematic representation of the paracrine effects of exosomes derived from SHED. Exosomes derived from osteogenic-conditioned SHED induce PDLSCs osteogenic differentiation via the activation of Wnt/β-catenin and BMP/Smad signaling pathways. 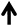: increase; all the other arrows indicate the direction of the flow; BMP2: Bone morphogenetic protein 2; PDLSCs: periodontal ligament stem cells; SHED: stem cells from exfoliated deciduous teeth; SMAD: small mother against decapentaplegic; Wnt3a: Wingless-related integration site family member 3A. See text for explanation [132].

Moreover, exosomes derived from osteogenic conditioned-SHED displayed higher osteogenic function in PDLSCs when compared to SHED without conditioning. Concerning the molecular mechanisms activated by SHED exosome, it was reported that Wnt3a and BMP2 up-regulation in exosomes accounted for the activation of Wnt/β-catenin and BMP/Smad signaling pathways in PDLSCs (Figure 5) [132].

Pivoraitė et al. first investigated immunomodulatory and anti-inflammatory function of exosomes derived from exfoliated deciduous teeth in an experimental animal model of paw inflammation (obtained by subcutaneous injection of 1% λ-carrageenan) [133]. Exosomes administration exerted strong anti-inflammatory effects, comparable with those of glucocorticoids. One potential mechanism by which exosomes achieved such result may involve the COX2, PLA2, and iNOS signaling pathways, however, to date, the supporting data appear poor.

Due to their embryological derivation by cranial neural crest cells, which are precursors of both neural and skeletal tissues, SHEDs is considered particularly suitable for the induction of neural differentiation as well as for neuroprotective action. The first evidence for neuroprotective action of SHED-derived exosomes was provided by [134], which tested the effects of exosomes and micro-vesicles derived from SHEDs on human dopaminergic neurons during oxidative stress induced by 6-OHDA. From the results it appeared that exosomes, but not micro-vesicles derived from SHEDs, suppressed 6-OHDA–induced apoptosis in dopaminergic neurons [134]. SHED-derived exosomes have been also tested for potential neuroprotective action after TBI. TBI is defined as damage to the brain caused by external mechanical force; at the beginning, TBI is characterized by pro-inflammatory events involving the release of inflammatory factors by microglia. Then, there is a transition of microglia from M1 to M2 with anti-inflammatory action, followed by tissue reparation. In some cases, pro-inflammatory events are not inhibited, so that tissue repair did not occur. It has been showed that stem cell therapy, by using neural stem cells could ameliorate traumatic brain injury (TBI) sequel, also via a direct shift of microglia from M1 to M2. Authors showed that administration of SHED-derived exosomes could promote functional motor recovery in rats after TBI, via a direct shift of microglia polarization from M1 to M2 [135].

### 6.5. Exosomes Derived from Gingival Mesenchymal Stem Cells (GMSC)

More recently, gingival mesenchymal stem cells (GMSC)-derived exosomes have also been tested for their regenerative potentials. In particular, the study of Zhang et al. tested the regenerative potential of small intestinal submucosa–extracellular matrix (SISECM) constructs both with gingival MSCs or their derivative exosomes in taste bud regeneration during tongue reconstruction [136]. From their results it appeared that SIS-ECM construct, in association with GMSCs, exerted an overall better beneficial effect on the reconstruction of lingual papillae structure and taste bud regeneration with respect to exosome/SIS-ECM constructs. These observations suggest that some non exosomal factors secreted by GMSCs may also play a role in facilitating taste bud regeneration, so in this case exosomes appeared to exert a lower action respect to the cell of origin [136].

### 6.6. Exosomes Derived from Other Oral Sources

Very recently, Zhang et al. tried to characterize exosomes in cells derived from Hertwig’s epithelial root sheath [137]. It’s clear that HERS is a transient structure assembled in the early period of the elongation of the root, so appeared difficult the use of the sample as experimental in vitro model. However, authors established an immortalized HERS cell line which resulted to be similar in morphology and characteristics to primary HERS cells, and could also induce odontogenic differentiation of dental papilla cells. HERS-derived exosomes are involved in this process.

Similarly, rat maxillary sinus mucosa-derived cells and mandibular periosteum-derived cells have been successfully used to isolate exosomes [138]. In both cases, exosomes exposure was able to enhance the proliferation, migration, and osteogenic differentiation of rat BMMSCs in vitro. In addition, exosomes-scaffold materials construct accelerated bone formation in rat femoral defects, confirming the in vivo action.

A detailed description of exosomes derived from stem cells from the apical papilla (SCAP) has been provided by [139]. They analyzed and compared PIWI-interacting RNAs (piRNAs) expression profiles in the exosomes of SCAP and the exosomes of BMMSCs, showing that they differ significantly. In particular, piRNAs are a new class of small ncRNAs forming RNA-induced silencing complexes by binding to PIWI-family subproteins [139]. According to the authors, the differentially expressed piRNAs could exert a specific role during the teeth morphogenesis and the formation of bone tissue, respectively. Functionally, exosomes derived from SCAP were able to promote BMMSC-based dentine-pulp complex regeneration when implanted subcutaneously into immunodeficient mice together with tooth fragments, BMMSCs, and scaffold. Moreover, exosomes derived from SCAP promoted the specific dentinogenesis of BMMSCs [140]. Exosomes also displayed anti-apoptotic activity in odontoblasts. Indeed, exosomes derived from odontoblasts exposed to high concentration of LPS exerted an anti-apoptotic action on odontoblast exposed to low concentration of LPS, suggesting a protective role during inflammation caused by caries [141].

The involvement of oral MSCs-derived exosomes in malignant transformation has also been showed [142]. Oral squamous cell carcinoma is one of the most common malignancy types globally, and in the majority of cases evolve from oral premalignant lesions, such as erythroplakia and oral leukoplakia. The role of MSCs and above all cancer cells-MSCs cross-talk in malignant transformation has not been elucidated, however many evidence suggested a key role for oral MSCs. Similar to physiological conditions, also in this case indirect cell-to-cell interactions mediated by the release of EVs have been suggested [143]. Li et al. showed that exosomes derived from erythroplakia and oral leukoplakia patients exerted a key role in promoting proliferation, migration and invasion in vitro of two cancer cell line. i.e., the DOK cell line (oral hyperplasia cell line) and the SCC15 cell line (oral carcinoma cell line), an action mediated by the up-regulation of exosomal miR-8485 [142].

**Table 1:** Recent key studies with methodology for oral MSCs-derived exosomes isolation.

Cell Type	Cell Source	Isolation Method	Reference
DPSCs, PDLSCs, SHED, SCAP	human	ultracentrifugation	[112,114,116,117,118,124,125,128,132,133,134,141]
SMCs (maxilla), PCs	rat	ultracentrifugation	[138]
DPSCs	human	Total exosome isolation reagent (Cat#4478359, Invitrogen, Carlsbad, CA, USA)	[115,120]
Hertwig’s epithelial root sheath-derived cells	rat	Total Exosome Isolation TM reagent (Life Technologies, Carlsbad, CA, USA)	[137]
DPSCs, PDLSCs, SHED, SCAP	human	ExoQuick-TC reagent (System Biosciences, Mountain View, CA, USA)	[113,123,126,129,135,139,140]
DPSCs	human	Exo-spin exosome isolation reagent (Cell Guidance, Cambridge, UK)	[119]
PDLSCs	human	PureExo^®^ exosome isolation kit (101Bio, Mountain View, CA, USA)	[127]
GMSC	human	N.A.	[136]
Oral mucosa MSCs	human	density gradient differential centrifugation	[142]

DPSCs: dental pulp stem cells; PDLSCs: periodontal ligament stem cells; SHEDs: stem cells from exfoliated deciduous teeth; GMSC: gingival mesenchymal stem cells; SMC: sinus mucosa-derived cells; PCs: mandibular periosteum-derived cells; SCAP: stem cells from apical papilla; N.A.: not available.

### 6.7. Exosomes as Promising Tool for MRONJ Management

Emerging role of exosomes as a tool for disease progression monitoring in humans and animals has been widely discussed in literature [144,145,146]. In the last decade, exosomes have been largely studied for their therapeutic potential, as well. In particular, biological properties of exosomes allow them to easily access to central nervous system, passing the blood brain barrier, so providing a vehicle for the delivery of drugs. The nanoscale size allows them to be rapidly uptake by cells, too. Exosomes can be engineered to target specific cell types or to carry specific cargo, via the stimulation or genetic alteration of parental cells. Moreover, the advantages of using exosomes in craniofacial tissue engineering and regeneration have been recently reviewed [147,148,149]. Regarding osteonecrosis of the jaw, we find just one paper investigating the effects of EVs administration to prevent MRONJ both in vivo and in vitro. Watanabe et al. showed that MSCs-derived exosomes are able to prevent zoledronate-induced senescence in cells populating alveolar socket microenvironment, so reducing the alterations reported in the MRONJ [150]. It’s noteworthy the role of oral MSCs-derived exosomes in immunomodulation, which might have a crucial role in managing the MRONJ.

## 7. Conclusions

Of course, the application of exosomes instead of MSCs may contribute to resolve risks of side effects associated to cell-based regenerative medicine, however many aspects attend to be resolved, among which the definition of standardized isolation and characterization protocol, the biological functions and molecular mechanisms of exosomes in MRONJ and their clinical translation.

## References

[1] Gnecchi M., Danieli P., Malpasso G., Ciuffreda M.C. (2016). Paracrine Mechanisms of Mesenchymal Stem Cells in Tissue Repair. Methods Mol. Biol..

[2] Barreca M.M., Cancemi P., Geraci F. (2020). Mesenchymal and Induced Pluripotent Stem Cells-Derived Extracellular Vesicles: The New Frontier for Regenerative Medicine?. Cells.

[3] Théry C., Witwer K.W., Aikawa E., Alcaraz M.J., Anderson J.D., Andriantsitohaina R., Antoniou A., Arab T., Archer F., Atkin-Smith G.K. (2018). Minimal information for studies of extracellular vesicles 2018 (MISEV2018): A position statement of the International Society for Extracellular Vesicles and update of the MISEV2014 guidelines. J. Extracell. Vesicles.

[4] Zhang L., Parot J., Hackley V.A., Turko I.V. (2020). Quantitative Proteomic Analysis of Biogenesis-Based Classification for Extracellular Vesicles. Proteomes.

[5] Zhang H., Lyden D. (2019). Asymmetric-flow field-flow fractionation technology for exomere and small extracellular vesicle separation and characterization. Nat. Protoc..

[6] Zhang H., Freitas D., Kim H.S., Fabijanic K., Li Z., Chen H., Mark M.T., Molina H., Martin A.B., Bojmar L. (2018). Identification of distinct nanoparticles and subsets of extracellular vesicles by asymmetric-flow field-flow fractionation. Nat. Cell Biol..

[7] Andrukhov O., Behm C., Blufstein A., Rausch-Fan X. (2019). Immunomodulatory properties of dental tissue-derived mesenchymal stem cells: Implication in disease and tissue regeneration. World J. Stem Cells.

[8] Khosla S., Burr D., Cauley J., Dempster D.W., Ebeling P.R., Felsenberg D., Gagel R.F., Gilsanz V., Guise T., Koka S. (2007). Bisphosphonate-associated osteonecrosis of the jaw: Report of a task force of the American Society for Bone and Mineral Research. J. Bone Miner. Res..

[9] Marx R.E. (2003). Pamidronate (Aredia) and zoledronate (Zometa) induced avascular necrosis of the jaws: A growing epidemic. J. Oral Maxillofac. Surg..

[10] Ruggiero S.L., Dodson T.B., Fantasia J., Goodday R., Aghaloo T., Mehrotra B., O’Ryan F., American Association of Oral and Maxillofacial Surgeons (2014). American Association of Oral and Maxillofacial Surgeons position paper on medication-related osteonecrosis of the jaw--2014 update. J. Oral Maxillofac. Surg..

[11] Brunello A., Saia G., Bedogni A., Scaglione D., Basso U. (2009). Worsening of osteonecrosis of the jaw during treatment with sunitinib in a patient with metastatic renal cell carcinoma. Bone.

[12] Taylor K.H., Middlefell L.S., Mizen K.D. (2010). Osteonecrosis of the jaws induced by anti-RANK ligand therapy. Br. J. Oral Maxillofac. Surg..

[13] Van Poznak C. (2010). Osteonecrosis of the jaw and bevacizumab therapy. Breast Cancer Res. Treat..

[14] Zarringhalam P., Brizman E., Shakib K. (2017). Medication-related osteonecrosis of the jaw associated with aflibercept. Br. J. Oral Maxillofac. Surg..

[15] Eguia A., Bagán-Debón L., Cardona F. (2020). Review and update on drugs related to the development of osteonecrosis of the jaw. Med. Oral Patol. Oral Cir. Bucal.

[16] Rogers M.J., Mönkkönen J., Munoz M.A. (2020). Molecular mechanisms of action of bisphosphonates and new insights into their effects outside the skeleton. Bone.

[17] Chiarella E., Codispoti B., Aloisio A., Cosentino E.G., Scicchitano S., Montalcini Y., Lico D., Morrone G., Mesuraca M., Bond H.M. (2020). Zoledronic acid inhibits the growth of leukemic MLL-AF9 transformed hematopoietic cells. Heliyon.

[18] Monkkonen H., Auriola S., Lehenkari P., Kellinsalmi M., Hassinen I.E., Vepsalainen J., Monkkonen J. (2006). A new endogenous ATP analog (ApppI) inhibits the mitochondrial adenine nucleotide translocase (ANT) and is responsible for the apoptosis induced by nitrogen-containing bisphosphonates. Br. J. Pharmacol..

[19] Dore R.K. (2011). The RANKL pathway and denosumab. Rheum. Dis. Clin. N. Am..

[20] Zaheer S., LeBoff M., Lewiecki E.M. (2015). Denosumab for the treatment of osteoporosis. Expert Opin. Drug Metab. Toxicol..

[21] Baron R., Ferrari S., Russell R.G.G. (2011). Denosumab and bisphosphonates: Different mechanisms of action and effects. Bone.

[22] Zirk M., Wenzel C., Buller J., Zöller J.E., Zinser M., Peters F. (2019). Microbial diversity in infections of patients with medication-related osteonecrosis of the jaw. Clin. Oral Investig..

[23] Ficarra G., Beninati F. (2007). Bisphosphonate-related osteonecrosis of the jaws: An update on clinical, pathological and management aspects. Head Neck Pathol..

[24] Pushalkar S., Li X., Kurago Z., Ramanathapuram L.V., Matsumura S., Fleisher K.E., Glickman R., Yan W., Li Y., Saxena D. (2014). Oral microbiota and host innate immune response in bisphosphonate-related osteonecrosis of the jaw. Int. J. Oral Sci..

[25] Zirk M., Kreppel M., Buller J., Pristup J., Peters F., Dreiseidler T., Zinser M., Zöller J.E. (2017). The impact of surgical intervention and antibiotics on MRONJ stage II and III—Retrospective study. J. Craniomaxillofac. Surg..

[26] Hinson A.M., Smith C.W., Siegel E.R., Stack B.C. (2014). Is bisphosphonate-related osteonecrosis of the jaw an infection? A histological and microbiological ten-year summary. Int. J. Dent..

[27] Russmueller G., Seemann R., Weiss K., Stadler V., Speiss M., Perisanidis C., Fuereder T., Willinger B., Sulzbacher I., Steininger C. (2016). The association of medication-related osteonecrosis of the jaw with *Actinomyces* spp. Infection. Sci. Rep..

[28] Zirk M., Zalesski A., Peters F., Dreiseidler T., Buller J., Kreppel M., Zöller J.E., Zinser M. (2018). Prevention and management of bacterial infections of the donor site of flaps raised for reconstruction in head and neck surgery. J. Craniomaxillofac. Surg..

[29] Dodson T.B., Raje N.S., Caruso P.A., Rosenberg A.E. (2008). Case records of the Massachusetts General Hospital. Case 9-2008. A 65-year-old woman with a nonhealing ulcer of the jaw. N. Engl. J. Med..

[30] Açil Y., Möller B., Niehoff P., Rachko K., Gassling V., Wiltfang J., Simon M.J. (2012). The cytotoxic effects of three different bisphosphonates in-vitro on human gingival fibroblasts, osteoblasts and osteogenic sarcoma cells. J. Craniomaxillofac. Surg..

[31] Marolt D., Cozin M., Vunjak-Novakovic G., Cremers S., Landesberg R. (2012). Effects of pamidronate on human alveolar osteoblasts in vitro. J. Oral Maxillofac. Surg..

[32] McLeod N.M., Moutasim K.A., Brennan P.A., Thomas G., Jenei V. (2014). In vitro effect of bisphosphonates on oral keratinocytes and fibroblasts. J. Oral Maxillofac Surg..

[33] De Colli M., Zara S., di Giacomo V., Patruno A., Marconi G.D., Gallorini M., Zizzari V.L., Tetè G., Cataldi A. (2015). Nitric oxide-mediated cytotoxic effect induced by zoledronic acid treatment on human gingival fibroblasts. Clin. Oral Investig..

[34] Manzano-Moreno F.J., Ramos-Torrecillas J., De Luna-Bertos E., Reyes-Botella C., Ruiz C., García-Martínez O. (2015). Nitrogen-containing bisphosphonates modulate the antigenic profile and inhibit the maturation and biomineralization potential of osteoblast-like cells. Clin. Oral Investig..

[35] Zara S., De Colli M., di Giacomo V., Zizzari V.L., Di Nisio C., Di Tore U., Salini V., Gallorini M., Tetè S., Cataldi A. (2015). Zoledronic acid atsubtoxic dose extendsosteoblastic stage span of primary human osteoblasts. Clin. Oral Investig..

[36] Jung J., Park J.S., Righesso L., Pabst A.M., Al-Nawas B., Kwon Y.D., Walter C. (2018). Effects of an oral bisphosphonate and three intravenous bisphosphonates on several cell types in vitro. Clin. Oral. Investig..

[37] Buxton I.L.O., Brunton L.L., Lazo J.S., Parker K.L. (2006). Pharmakokinetics and pharmacodynamics: The dynamics of drug absorption, distribution, action, and elimination. Goodman and Gilman’s the Pharmacological Basis of Therapeutics.

[38] Coxon F.P., Thompson K., Roelofs A.J., Ebetino F.H., Rogers M.J. (2008). Visualizing mineral binding and uptake of bisphosphonate by osteoclasts and non-resorbing cells. Bone.

[39] Okada S., Kiyama T., Sato E., Tanaka Y., Oizumi T., Kuroishi T., Takahashi T., Sasaki K., Sugawara S., Endo Y. (2013). Inhibition of phosphate transporters ameliorates the inflammatory and necrotic side effects of the nitrogen-containing bisphosphonate zoledronate in mice. Tohoku J. Exp. Med..

[40] Thompson K., Rogers M.J., Coxon F.P., Crockett J.C. (2006). Cytosolic entry of bisphosphonate drugs requires acidification of vesicles after fluid-phase endocytosis. Mol. Pharmacol..

[41] Junankar S., Shay G., Jurczyluk J., Ali N., Down J., Pocock N., Parker A., Nguyen A., Sun S., Kashemirov B. (2015). Real-time intravital imaging establishes tumor-associated macrophages as the extraskeletal target of bisphosphonate action in cancer. Cancer Discov..

[42] Ikebe T. (2013). Pathophysiology of BRONJ: Drug-related osteoclastic disease of the jaw. Oral Sci. Int..

[43] Oizumi T., Yamaguchi K., Funayama H., Kuroishi T., Kawamura H., Sugawara S., Endo Y. (2009). Necrotic actions of nitrogen-containing bisphosphonates and their inhibition by clodronate, a non-nitrogen-containing bisphosphonate in mice: Potential for utilization of clodronate as a combination drug with a nitrogen-containing bisphosphonate. Basic Clin. Pharmacol. Toxicol..

[44] Oizumi T., Funayama H., Yamaguchi K., Yokoyama M., Takahashi H., Yamamoto M., Kuroishi T., Kumamoto H., Sasaki K., Kawamura H. (2010). Inhibition of necrotic actions of nitrogen-containing bisphosphonates (NBPs) and their elimination from bone by etidronate (a non-NBP): A proposal for possible utilization of etidronate as a substitution drug for NBPs. J. Oral Maxillofac. Surg..

[45] Kiyama T., Tsuchiya M., Okada S., Oizumi T., Yamaguchi K., Sasaki K., Sugawara S., Endo Y. (2016). Phosphonocarboxylates Can Protect Mice against the Inflammatory and Necrotic Side Effects of Nitrogen-Containing Bisphosphonates by Inhibiting Their Entry into Cells via Phosphate Transporters. Biol. Pharm. Bull..

[46] Endo Y., Nakamura M., Kikuchi T., Shinoda H., Takeda Y., Nitta Y., Kumagai K. (1993). Aminoalkylbisphosphonates, potent inhibitors of bone resorption, induce a prolonged stimulation of histamine synthesis and increase macrophages, granulocytes, and osteoclasts in vivo. Calcif. Tissue Int..

[47] Roelofs A.J., Thompson K., Ebetino F.H., Rogers M.J., Coxon F.P. (2010). Bisphosphonates: Molecular mechanisms of action and effects on bone cells, monocytes and macrophages. Curr. Pharm. Des..

[48] Kimachi K., Kajiya H., Nakayama S., Ikebe T., Okabe K. (2011). Zoledronic acid inhibits RANK expression and migration of osteoclast precursors during osteoclastogenesis. Naunyn-Schmiedeberg’s Arch. Pharmacol..

[49] Chen Y.J., Clifford Chao K.S., Yang Y.C., Hsu M.L., Lin C.P., Chen Y.Y. (2009). Zoledronic acid, an aminobisphosphonate, modulates differentiation and maturation of human dendritic cells. Immunopharmacol. Immunotoxicol..

[50] Comito G., Pons Segura C., Taddei M.L., Lanciotti M., Serni S., Morandi A., Chiarugi P., Giannoni E. (2017). Zoledronic acid impairs stromal reactivity by inhibiting M2-macrophages polarization and prostate cancer-associated fibroblasts. Oncotarget.

[51] Zhu W., Xu R., Du J., Fu Y., Li S., Zhang P., Liu L., Jiang H. (2019). Zoledronic acid promotes TLR-4-mediated M1 macrophage polarization in bisphosphonate-related osteonecrosis of the jaw. FASEB J..

[52] Lee K.H., Kang T.B. (2019). The Molecular Links between Cell Death and Inflammasome. Cells.

[53] Shikama Y., Nagai Y., Okada S., Oizumi T., Shimauchi H., Sugawara S., Endo Y. (2010). Pro-IL-1β accumulation in macrophages by alendronate and its prevention by clodronate. Toxicol. Lett..

[54] Yang X., Xu X., Chen J., Wang Q., Wang G., Ai X., Wang X., Pan J. (2020). Zoledronic acid regulates the synthesis and secretion of IL-1β through Histone methylation in macrophages. Cell Death Discov..

[55] Endo Y., Kumamoto H., Nakamura M., Sugawara S., Takano-Yamamoto T., Sasaki K., Takahashi T. (2017). Underlying Mechanisms and Therapeutic Strategies for Bisphosphonate-Related Osteonecrosis of the Jaw (BRONJ). Biol. Pharm. Bull..

[56] Kunzmann V., Bauer E., Feurle J., Weissinger F., Tony H.P., Wilhelm M. (2000). Stimulation of T Cells by aminobisphosphonates and induction of antiplasma cell activity in multiple myeloma. Blood.

[57] Thompson K., Rogers M.J. (2004). Statins prevent bisphosphonate-induced γ,δ-T-cell proliferation and activation in vitro. J. Bone Miner. Res..

[58] Fiore F., Castella B., Nuschak B., Bertieri R., Mariani S., Bruno B., Pantaleoni F., Foglietta M., Boccadoro M., Massaia M. (2007). Enhanced ability of dendritic cells to stimulate innate and adaptive immunity on short-term incubation with zoledronic acid. Blood.

[59] Roelofs A.J., Jauhiainen M., Mönkkönen H., Rogers M.J., Mönkkönen J., Thompson K. (2009). Peripheral blood monocytes are responsible for γδ T cell activation induced by zoledronic acid through accumulation of IPP/DMAPP. Br. J. Haematol..

[60] Castella B., Kopecka J., Sciancalepore P., Mandili G., Foglietta M., Mitro N., Caruso D., Novelli F., Riganti C., Massaia M. (2017). The ATP-binding cassette transporter A1 regulates phosphoantigen release and Vγ9Vδ2 T cell activation by dendritic cells. Nat. Commun..

[61] Riganti C., Castella B., Massaia M. (2018). ABCA1, apoA-I, and BTN3A1: A Legitimate Ménage à Trois in DendriticCells. Front. Immunol..

[62] Kalyan S., Quabius E.S., Wiltfang J., Mönig H., Kabelitz D. (2013). Can peripheral blood γδ T Cells predict osteonecrosis of the jaw? An immunological perspective on the adverse drug effects of aminobisphosphonate therapy. J. Bone Miner. Res..

[63] Kalyan S. (2016). It may be Inflammatory, but Some T Cells Are Innately Healing to the Bone. J. Bone Miner. Res..

[64] Movila A., Mawardi H., Nishimura K., Kiyama T., Egashira K., Kim J.Y., Villa A., Sasaki H., Woo S.B., Kawai T. (2016). Possible pathogenic engagement of soluble Semaphorin 4D produced by γδT cells in medication-related osteonecrosis of the jaw (MRONJ). Biochem. Biophys. Res. Commun..

[65] Van Acker H.H., Anguille S., Willemen Y., Smits E.L., Van Tendeloo V.F. (2016). Bisphosphonates for cancer treatment: Mechanisms of action and lessons from clinical trials. Pharmacol. Ther..

[66] Liu H., Wang S.H., Chen S.C., Chen C.Y., Lin T.M. (2019). Zoledronic acid blocks the interaction between breast cancer cells and regulatory T-cells. BMC Cancer.

[67] Rossini M., Adami S., Viapiana O., Fracassi E., Ortolani R., Vella A., Zanotti R., Tripi G., Idolazzi L., Gatti D. (2012). Long-term effects of amino-bisphosphonates on circulating γδ T cells. Calcif. Tissue Int..

[68] Hagelauer N., Ziebart T., Pabst A.M., Walter C. (2015). Bisphosphonates inhibit cell functions of HUVECs, fibroblasts and osteogenic cells via inhibition of protein geranylgeranylation. Clin. Oral Investig..

[69] Tseng H.C., Kanayama K., Kaur K., Park S.H., Park S., Kozlowska A., Sun S., McKenna C.E., Nishimura I., Jewett A. (2015). Bisphosphonate-induced differential modulation of immune cell function in gingiva and bone marrow in vivo: Role in osteoclast-mediated NK cell activation. Oncotarget.

[70] Walter C., Pabst A., Ziebart T., Klein M., Al-Nawas B. (2011). Bisphosphonates affect migration ability and cell viability of HUVEC, fibroblasts and osteoblasts in vitro. Oral Dis..

[71] Cackowski F.C., Anderson J.L., Patrene K.D., Choksi R.J., Shapiro S.D., Windle J.J., Blair H.C., Roodman G.D. (2010). Osteoclasts are important for bone angiogenesis. Blood.

[72] Ziebart T., Ziebart J., Gauss L., Pabst A., Ackermann M., Smeets R., Konerding M.A., Walter C. (2013). Investigation of inhibitory effects on EPC-mediated neovascularization by different bisphosphonates for cancer therapy. Biomed. Rep..

[73] Ohlrich E.J., Coates D.E., Cullinan M.P., Milne T.J., Zafar S., Zhao Y., Duncan W.D., Seymour G.J. (2016). The bisphosphonate zoledronic acid regulates key angiogenesis-related genes in primary human gingival fibroblasts. Arch. Oral Biol..

[74] Basi D.L., Lee S.W., Helfman S., Mariash A., Lunos S.A. (2010). Accumulation of VEGFR2 in zoledronic acid-treated endothelial cells. Mol. Med. Rep..

[75] Wehrhan F., Amann K., Möbius P., Weber M., Preidl R., Ries J., Stockmann P. (2015). BRONJ-related jaw bone is associated with increased Dlx-5 and suppressed osteopontin-implication in the site-specific alteration of angiogenesis and bone turnover by bisphosphonates. Clin. Oral Investig..

[76] Sharma D., Hamlet S.M., Petcu E.B., Ivanovski S. (2016). The effect of bisphosphonates on the endothelial differentiation of mesenchymal stem cells. Sci. Rep..

[77] Bermúdez-Bejarano E.B., Serrera-Figallo M.Á., Gutiérrez-Corrales A., Romero-Ruiz M.M., Castillo-de-Oyagüe R., Gutiérrez-Pérez J.L., Torres-Lagares D. (2017). Prophylaxis and antibiotic therapy in management protocols of patients treated with oral and intravenous bisphosphonates. J. Clin. Exp. Dent..

[78] Ristow O., Otto S., Troeltzsch M., Hohlweg-Majert B., Pautke C. (2015). Treatment perspectives for medication-related osteonecrosis of the jaw (MRONJ). J. Craniomaxillofac. Surg..

[79] Kang S.H., Won Y.J., Kim M.K. (2018). Surgical treatment of stage 2 medication-related osteonecrosis of the jaw compared to osteomyelitis. Cranio.

[80] Giudice A., Barone S., Diodati F., Antonelli A., Nocini R., Cristofaro M.G. (2020). Can Surgical Management Improve Resolution of Medication-Related Osteonecrosis of the Jaw at Early Stages? A Prospective Cohort Study. J. Oral Maxillofac. Surg..

[81] Vescovi P., Merigo E., Fornaini C., Rocca J.P., Nammour S. (2012). Thermal increase in the oral mucosa and in the jawbone during Nd:YAG laser applications. Ex vivo study. Med. Oral Patol. Oral Cir. Bucal.

[82] Şahin O., Odabaşi O., Ekmekcioğlu C. (2019). Ultrasonic Piezoelectric Bone Surgery Combined With Leukocyte and Platelet-Rich Fibrin and Pedicled Buccal Fat Pad Flap in Denosumab-Related Osteonecrosis of the Jaw. J. Craniofac. Surg..

[83] Giudice A., Bennardo F., Barone S., Antonelli A., Figliuzzi M.M., Fortunato L. (2018). Can Autofluorescence Guide Surgeons in the Treatment of Medication-Related Osteonecrosis of the Jaw? A Prospective Feasibility Study. J. Oral Maxillofac. Surg..

[84] Voss P.J., Matsumoto A., Alvarado E., Schmelzeisen R., Duttenhöfer F., Poxleitner P. (2017). Treatment of stage II medication-related osteonecrosis of the jaw with necrosectomy and autologous bone marrow mesenchymal stem cells. Odontology.

[85] Giudice A., Barone S., Giudice C., Bennardo F., Fortunato L. (2018). Can platelet-rich fibrin improve healing after surgical treatment of medication-related osteonecrosis of the jaw? A pilot study. Oral Surg. Oral Med. Oral Pathol. Oral Radiol..

[86] Bennardo F., Bennardo L., Del Duca E., Patruno C., Fortunato L., Giudice A., Nisticò S.P. (2020). Autologous platelet-rich fibrin injections in the management of facial cutaneous sinus tracts secondary to medication-related osteonecrosis of the jaw. Dermatol. Ther..

[87] Fortunato L., Bennardo F., Buffone C., Giudice A. (2020). Is the application of platelet concentrates effective in the prevention and treatment of medication-related osteonecrosis of the jaw? A systematic review. J. Craniomaxillofac. Surg..

[88] Kaibuchi N., Iwata T., Onizuka S., Yano K., Tsumanuma Y., Yamato M., Okano T., Ando T. (2019). Allogeneic multipotent mesenchymal stromal cell sheet transplantation promotes healthy healing of wounds caused by zoledronate and dexamethasone in canine mandibular bones. Regener. Ther..

[89] Cella L., Oppici A., Arbasi M., Moretto M., Piepoli M., Vallisa D., Zangrandi A., Di Nunzio C., Cavanna L. (2011). Autologous bone marrow stem cell intralesional transplantation repairing bisphosphonate related osteonecrosis of the jaw. Head Face Med..

[90] Di Vito A., Chiarella E., Baudi F., Scardamaglia P., Antonelli A., Giudice D., Barni T., Fortunato L., Giudice A. (2020). Dose-Dependent Effects of Zoledronic Acid on Human Periodontal Ligament Stem Cells: An In Vitro Pilot Study. Cell Transplant..

[91] Li M., Yu Y., Shi Y., Zhou Y., Zhang W., Hua H., Ge J., Zhang Z., Ye D., Yang C. (2020). Decreased Osteogenic Ability of Periodontal Ligament Stem Cells Leading to Impaired Periodontal Tissue Repair in BRONJ Patients. Stem Cells Dev..

[92] Şahin O., Tatar B., Ekmekcioğlu C., Aliyev T., Odabaşı O. (2020). Prevention of medication related osteonecrosis of the jaw after dentoalveolar surgery: An institution’s experience. J. Clin. Exp. Dent..

[93] Chang J., Hakam A.E., McCauley L.K. (2018). Current Understanding of the Pathophysiology of Osteonecrosis of the Jaw. Curr. Osteoporos. Rep..

[94] Weston L.A., Thiery J.P. (2015). Pentimento: Neural Crest and the origin of mesectoderm. Dev. Biol..

[95] Noden D.M., Trainor P.A. (2005). Relations and interactions between cranial mesoderm and neural crest populations. J. Anat..

[96] Zhu Y., Zhang P., Gu R.L., Liu Y.S., Zhou Y.S. (2018). Origin and Clinical Applications of Neural Crest-Derived Dental Stem Cells. Chin. J. Dent. Res..

[97] Chiarella E., Aloisio A., Scicchitano S., Lucchino V., Montalcini Y., Galasso O., Greco M., Gasparini G., Mesuraca M., Bond H.M. (2018). ZNF521 Represses Osteoblastic Differentiation in Human Adipose-Derived Stem Cells. Int. J. Mol. Sci..

[98] Di Vito A., Giudice A., Chiarella E., Malara N., Bennardo F., Fortunato L. (2019). In Vitro Long-Term Expansion and High Osteogenic Potential of Periodontal Ligament Stem Cells: More Than a Mirage. Cell Transplant..

[99] Johnstone R.M., Adam M., Hammond J.R., Orr L., Turbide C. (1987). Vesicle formation during reticulocyte maturation. Association of plasma membrane activities with released vesicles (exosomes). J. Biol. Chem..

[100] Conde-Vancells J., Rodriguez-Suarez E., Gonzalez E., Berisa A., Gil D., Embade N., Valle M., Luka Z., Elortza F., Wagner C. (2010). Candidate biomarkers in exosome-like vesicles purified from rat and mouse urine samples. Proteom. Clin. Appl..

[101] Palmulli R., van Niel G. (2018). To be or not to be… secreted as exosomes, a balance finely tuned by the mechanisms of biogenesis. Essays Biochem..

[102] Kalra H., Simpson R.J., Ji H., Aikawa E., Altevogt P., Askenase P., Bond V.C., Borràs F.E., Breakefield X., Budnik V. (2012). Vesiclepedia: A compendium for extracellular vesicles with continuous community annotation. PLoS Biol..

[103] Mathivanan S., Simpson R.J. (2009). ExoCarta: A compendium of exosomal proteins and RNA. Proteomics.

[104] Kalluri R., LeBleu V.S. (2020). The biology, function, and biomedical applications of exosomes. Science.

[105] Willms E., Johansson H.J., Mäger I., Lee Y., Blomberg K.E., Sadik M., Alaarg A., Smith C.I., Lehtiö J., El Andaloussi S. (2016). Cells release subpopulations of exosomes with distinct molecular and biological properties. Sci. Rep..

[106] Pegtel D.M., Gould S.J. (2019). Exosomes. Annu. Rev. Biochem..

[107] Booth A.M., Fang Y., Fallon J.K., Yang J.M., Hildreth J.E., Gould S.J. (2006). Exosomes and HIV Gag bud from endosome-like domains of the T cell plasma membrane. J. Cell Biol..

[108] Scott C.C., Vacca F., Gruenberg J. (2014). Endosome maturation, transport and functions. Semin. Cell Dev. Biol..

[109] Ba Q., Yang G. (2017). Intracellular organelle networks: Understanding their organization and communication through systems-level modeling and analysis. Front. Biol..

[110] Van Niel G., D’Angelo G., Raposo G. (2018). Shedding light on the cell biology of extracellular vesicles. Nat. Rev. Mol. Cell Biol..

[111] Skryabin G.O., Komelkov A.V., Savelyeva E.E., Tchevkina E.M. (2020). Lipid Rafts in Exosome Biogenesis. Biochemistry (Moscow).

[112] Altanerova U., Benejova K., Altanerova V., Tyciakova S., Rychly B., Szomolanyi P., Ciampor F., Cihova M., Repiska V., Ondicova K. (2016). Dental pulp mesenchymal stem/stromal cells labeled with iron sucrose release exosomes and cells applied intra-nasally migrate to intracerebral glioblastoma. Neoplasma.

[113] Huang C.C., Narayanan R., Alapati S., Ravindran S. (2016). Exosomes as biomimetic tools for stem cell differentiation: Applications in dental pulp tissue regeneration. Biomaterials.

[114] Gonzalez-King H., García N.A., Ontoria-Oviedo I., Ciria M., Montero J.A., Sepúlveda P. (2017). Hypoxia Inducible Factor-1α Potentiates Jagged 1-Mediated Angiogenesis by Mesenchymal Stem Cell-Derived Exosomes. Stem Cells.

[115] Venugopal C., Rai K.S., Pinnelli V.B., Kutty B.M., Dhanushkodi A. (2018). Neuroprotection by Human Dental Pulp Mesenchymal Stem Cells: From Billions to Nano. Curr. Gene Ther..

[116] Xian X., Gong Q., Li C., Guo B., Jiang H. (2018). Exosomes with Highly Angiogenic Potential for Possible Use in Pulp Regeneration. J. Endod..

[117] Li J., Ju Y., Liu S., Fu Y., Zhao S. (2019). Exosomes derived from lipopolysaccharide-preconditioned human dental pulp stem cells regulate Schwann cell migration and differentiation. Connect. Tissue Res..

[118] Ji L., Bao L., Gu Z., Zhou Q., Liang Y., Zheng Y., Xu Y., Zhang X., Feng X. (2019). Comparison of immunomodulatory properties of exosomes derived from bone marrow mesenchymal stem cells and dental pulp stem cells. Immunol. Res..

[119] Hu X., Zhong Y., Kong Y., Chen Y., Feng J., Zheng J. (2019). Lineage-specific exosomes promote the odontogenic differentiation of human dental pulp stem cells (DPSCs) through TGFβ1/smads signaling pathway via transfer of microRNAs. Stem Cell Res. Ther..

[120] Ivica A., Ghayor C., Zehnder M., Valdec S., Weber F.E. (2020). Pulp-Derived Exosomes in a Fibrin-Based Regenerative Root Filhuangng Material. J. Clin. Med..

[121] Bernaudo F., Monteleone F., Mesuraca M., Krishnan S., Chiarella E., Scicchitano S., Cuda G., Morrone G., Bond H.M., Gaspari M. (2015). Validation of a novel shotgun proteomic workflow for the discovery of protein-protein interactions: Focus on ZNF521. J. Proteome Res..

[122] Couve E., Lovera M., Suzuki K., Schmachtenberg O. (2018). Schwann Cell Phenotype Changes in Aging Human Dental Pulp. J. Dent. Res..

[123] Rajan T.S., Giacoppo S., Diomede F., Ballerini P., Paolantonio M., Marchisio M., Piattelli A., Bramanti P., Mazzon E., Trubiani O. (2016). The secretome of periodontal ligament stem cells from MS patients protects against EAE. Sci. Rep..

[124] Zheng Y., Dong C., Yang J., Jin Y., Zheng W., Zhou Q., Liang Y., Bao L., Feng G., Ji J. (2019). Exosomal microRNA-155-5p from PDLSCs regulated Th17/Treg balance by targeting sirtuin-1 in chronic periodontitis. J. Cell. Physiol..

[125] Zhao L.R., Mao J.Q., Zhao B.J., Chen J. (2019). Isolation and biological characteristics of exosomes derived from periodontal ligament stem cells. Shanghai Kou Qiang Yi Xue.

[126] Zhao M., Dai W., Wang H., Xue C., Feng J., He Y., Wang P., Li S., Bai D., Shu R. (2019). Periodontal ligament fibroblasts regulate osteoblasts by exosome secretion induced by inflammatory stimuli. Arch. Oral Biol..

[127] Wang Z., Maruyama K., Sakisaka Y., Suzuki S., Tada H., Suto M., Saito M., Yamada S., Nemoto E. (2019). Cyclic Stretch Force Induces Periodontal Ligament Cells to Secrete Exosomes That Suppress IL-1β Production Through the Inhibition of the NF-κB Signaling Pathway in Macrophages. Front. Immunol..

[128] Zhang Z., Shuai Y., Zhou F., Yin J., Hu J., Guo S., Wang Y., Liu W. (2020). PDLSCs Regulate Angiogenesis of Periodontal Ligaments via VEGF Transferred by Exosomes in Periodontitis. Int. J. Med. Sci..

[129] SoundaraRajan T., Giacoppo S., Diomede F., Bramanti P., Trubiani O., Mazzon E. (2017). Human periodontal ligament stem cells secretome from multiple sclerosis patients suppresses NALP3 inflammasome activation in experimental autoimmune encephalomyelitis. Int. J. Immunopathol. Pharmacol..

[130] Zhu Y., Huang M., Zhao Y., Pei Y., Wang Y., Wang L., He T., Zhou F., Zeng X. (2020). Local functional connectivity of patients with acute and remitting multiple sclerosis: A Kendall’s coefficient of concordance- and coherence-regional homogeneity study. Medicine.

[131] Martinez Saez D., Tetsuo Sasaki R., da Costa Neves A., da Silva M.C. (2016). Stem Cells from Human Exfoliated Deciduous Teeth: A Growing Literature. Cells Tissues Organs.

[132] Wang M., Li J., Ye Y., He S., Song J. (2020). SHED-derived conditioned exosomes enhance the osteogenic differentiation of PDLSCs via Wnt and BMP signaling in vitro. Differentiation.

[133] Pivoraitė U., Jarmalavičiūtė A., Tunaitis V., Ramanauskaitė G., Vaitkuvienė A., Kašėta V., Biziulevičienė G., Venalis A., Pivoriūnas A. (2015). Exosomes from Human Dental Pulp Stem Cells Suppress Carrageenan-Induced Acute Inflammation in Mice. Inflammation.

[134] Jarmalavičiūtė A., Tunaitis V., Pivoraitė U., Venalis A., Pivoriūnas A. (2015). Exosomes from dental pulp stem cells rescue human dopaminergic neurons from 6-hydroxy-dopamine-induced apoptosis. Cytotherapy.

[135] Li Y., Yang Y.Y., Ren J.L., Xu F., Chen F.M., Li A. (2017). Exosomes secreted by stem cells from human exfoliated deciduous teeth contribute to functional recovery after traumatic brain injury by shifting microglia M1/M2 polarization in rats. Stem Cell Res. Ther..

[136] Zhang Y., Shi S., Xu Q., Zhang Q., Shanti R.M., Le A.D. (2019). SIS-ECM Laden with GMSC-Derived Exosomes Promote Taste Bud Regeneration. J. Dent. Res..

[137] Zhang S., Yang Y., Jia S., Chen H., Duan Y., Li X., Wang S., Wang T., Lyu Y., Chen G. (2020). Exosome-like vesicles derived from Hertwig’s epithelial root sheath cells promote the regeneration of dentin-pulp tissue. Theranostics.

[138] Sun R., Xu S., Wang Z. (2019). Rat sinus mucosa- and periosteum-derived exosomes accelerate osteogenesis. J. Cell. Physiol..

[139] Wang A., Liu J., Zhuang X., Yu S., Zhu S., Liu Y., Chen X. (2020). Identification and Comparison of piRNA Expression Profiles of Exosomes Derived from Human Stem Cells from the Apical Papilla and Bone Marrow Mesenchymal Stem Cells. Stem Cells Dev..

[140] Zhuang X., Ji L., Jiang H., Liu Y., Liu X., Bi J., Zhao W., Ding Z., Chen X. (2020). Exosomes Derived from Stem Cells from the Apical Papilla Promote Dentine-Pulp Complex Regeneration by Inducing Specific Dentinogenesis. Stem Cells Int..

[141] Wang H.S., Yang F.H., Wang Y.J., Pei F., Chen Z., Zhang L. (2019). Odontoblastic Exosomes Attenuate Apoptosis in Neighboring Cells. J. Dent. Res..

[142] Li W., Han Y., Zhao Z., Ji X., Wang X., Jin J., Wang Q., Guo X., Cheng Z., Lu M. (2019). Oral mucosal mesenchymal stem cell-derived exosomes: A potential therapeutic target in oral premalignant lesions. Int. J. Oncol..

[143] Melzer C., von der Ohe J., Hass R. (2018). Concise Review: Crosstalk of Mesenchymal Stroma/Stem-Like Cells with Cancer Cells Provides Therapeutic Potential. Stem Cells..

[144] Loria A.D., Dattilo V., Santoro D., Guccione J., De Luca A., Ciaramella P., Pirozzi M., Iaccino E. (2020). Expression of Serum Exosomal miRNA 122 and Lipoprotein Levels in Dogs Naturally Infected by Leishmaniainfantum: A Preliminary Study. Animals.

[145] Maisano D., Mimmi S., Russo R., Fioravanti A., Fiume G., Vecchio E., Nisticò N., Quinto I., Iaccino E. (2020). Uncovering the Exosomes Diversity: A Window of Opportunity for Tumor Progression Monitoring. Pharmaceuticals.

[146] Iaccino E., Mimmi S., Dattilo V., Marino F., Candeloro P., Di Loria A., Marimpietri D., Pisano A., Albano F., Vecchio E. (2017). Monitoring multiple myeloma by idiotype-specific peptide binders of tumor-derivedexosomes. Mol. Cancer.

[147] Holliday L.S., McHugh K.P., Zuo J., Aguirre J.I., Neubert J.K., Rody W.J. (2017). Exosomes: Novel regulators of bone remodelling and potential therapeutic agents for orthodontics. Orthod. Craniofac. Res..

[148] Cooper L.F., Ravindran S., Huang C.C., Kang M. (2020). A Role for Exosomes in Craniofacial Tissue Engineering and Regeneration. Front. Physiol..

[149] Peng Q., Yang J.Y., Zhou G. (2020). Emerging functions and clinical applications of exosomes in human oral diseases. Cell Biosci..

[150] Watanabe J., Sakai K., Urata Y., Toyama N., Nakamichi E., Hibi H. (2020). Extracellular Vesicles of Stem Cells to Prevent BRONJ. J. Dent. Res..

